# Distribution Characteristics and Seasonal Variation of Soil Nutrients in the Mun River Basin, Thailand

**DOI:** 10.3390/ijerph15091818

**Published:** 2018-08-23

**Authors:** Zhonghe Zhao, Gaohuan Liu, Qingsheng Liu, Chong Huang, He Li, Chunsheng Wu

**Affiliations:** 1 State Key Laboratory of Resources and Environmental Information System, Institute of Geographic Sciences and Natural Resources Research, Chinese Academy of Sciences, Beijing 100101, China; zhaozh.16b@igsnrr.ac.cn (Z.Z.); huangch@lreis.ac.cn (C.H.); lih@lreis.ac.cn (H.L.); wuchsh0118@163.com (C.W.); 2 University of Chinese Academy of Sciences, Beijing 100049, China

**Keywords:** soil nutrient, seasonal variation, spatial variation, spatial pattern, Mun River Basin

## Abstract

Based on soil sampling data from the dry season and the rainy season, the spatial heterogeneity and spatial pattern of soil nutrients in the Mun River Basin, Thailand, were studied and the seasonal variation in soil nutrients was analyzed using classical statistical methods and geostatistical methods. The soil nutrient content in the Mun Basin showed moderate and strong variations, and the descending order of soil variability was as follows: available phosphorous (AP) > electric conductivity (EC) > soil organic matter (SOM) > total nitrogen (TN) > pH value in the dry season, with AP showing strong variation, and EC > AP > SOM > TN > pH in the rainy season, with EC showing strong variation. Different soil nutrients and different soil properties had different spatial variation characteristics, and their corresponding best-fitting models were also different. Based on the nugget (C_0_), sill (C_0_ + C), and range (A), spatial analysis was performed for the soil nutrients, pH, and EC in the dry season and in the rainy season. Analysis based on kriging spatial interpolation data showed that pH, SOM, TN, and EC had convex or concave distributions, whereas AP had a patchy distribution. Terrain, vegetation, and human disturbance are the main factors that contribute to the differences in the soil nutrient pattern of the Mun River Basin.

## 1. Introduction

Changes in land-use types and the excessive use of fertilizers (such as nitrogen (N) and phosphorus (P) fertilizers) in agriculture have changed soil nutrient content and soil properties. As important natural resources, soil ecosystems maintain the structural stability and the development of terrestrial ecosystems and promote ecological processes such as the recycling of underground ecosystems and nutrients [1,2]. Soil nutrient status is an important indicator of fertility because it not only coordinates and supplies the nutrients needed for plant growth but also promotes the decomposition of soil humus and the biogeochemical cycle [3]. In addition, soil nutrients can reflect the quality of the soil and the health status of the ecosystem [4,5]. Plant diversity affects the structural composition of vegetation and is an important indicator in maintaining the stable growth of vegetation [6,7]. The aboveground vegetation and the underground soil ecosystem complement and influence each other, with the soil system providing a large number of nutrients for the stable growth of vegetation and in turn promoting the structural stability of the soil system [6,8]. Therefore, spatial patterns of soil nutrients are a hot spot in ecological research and also provide an important theoretical basis for the restoration and reconstruction of vegetation.

Soil is a natural complex that has particularly complex morphology and evolution. To quantitatively describe soil’s temporal and spatial variations is difficult, especially for the quantitative description of its spatial variation. The introduction of geostatistical methods into soil analysis has promoted the development of this research direction. The use of geostatistics to study the spatial variability of soil properties involves a wide range of areas, including soil physicochemical properties, soil biological properties, soil heavy metal pollution, and even soil remediation.

In 1970s, research on the spatial variability of soil physical properties in North America and Western Europe resulted in the development of processing methods that take soil moisture parameters as random variables and the development of stochastic models for soil water movement. In the 1980s, based on analysis of the spatial variation of soil properties, Burgess et al. [9] introduced, a new soil prediction and simulation techniques, which greatly promoted the development of research. Andrew et al. [10] used remote sensing and field measurements to study the spatial variation of soil water in the Tarrawarra River Basin. Liang and Yao [11] studied the spatial variation characteristics of the main physical properties of hilly red soil and found that the co-kriging method is a superb and feasible method for using soil samples to estimate soil properties. Wang et al. [12] studied the spatial structure characteristics and the seasonal variation pattern of soil moisture in small basins in the loess hills in China, extending the research methods from the ordinary kriging method to the co-kriging method and the indicator kriging method and using geostatistics in combination with other methods (such as fractal theory) in an attempt to study the spatial variation of soil physical properties.

The use of geostatistics to study the spatial heterogeneity of soil chemical properties mainly focused on the study of soil nutrients. Until now, a large number of scholars had studied the spatial variability of soil nutrients [13]. Wang and Zhong collected tillage-layer soil samples and studied the spatial variability of the soil organic matter (SOM) [14]. Zhou et al. [15] collected soil samples and analyzed the spatial variability of soil AP and available potassium (AK) for the surface layers under grazing conditions. Using traditional statistical methods and geostatistical methods for analysis, two trends/scenarios exist in the calculation of semivariance. In the first scenario, no spatial structure is present, and statistical analysis can be performed using traditional statistics. In the second scenario, a spatial variation exists in the structure, and linear or curve-fitting equations can be used to estimate the semivariance parameters for several elements according to their north–south, east–west, and full range spacing. Zhang et al. [16] and Qin et al. [17] explored the intrinsic assumptions, semivariance functions, and kriging interpolation, and other theoretical problems, and quantified the spatial variation of the soil nutrients in large-scale regions. Yang and Zhang [18] studied the spatial variation of the AP and AK in a cotton field; Guo et al. [13] used GIS methods to study the mass fractions of alkali-hydrolyzable N, total N (TN), AK, AP, and SOM in the soil surface layer (0–20 cm) in Zunhua City, China, and demonstrated that GIS could combine attributive data with geographic data for system variables to perform geostatistical analysis on a large scale.

Chinese scholars [19] studied the differences in soil nutrients status under different land use patterns, they used methods of geostatistical analysis and GIS to study the content and spatial correlation of soil nutrients under two land use types of kiwifruit orchard and wheat-maize rotation farmland in Zhouzhi County, Shaanxi Province. The characteristics of spatial variability and distribution patterns of soil nutrients were also analyzed. The results showed that the content of organic matter, alkali-hydrolysable nitrogen, available phosphorus, pH value, and readily available potassium wear quite different in kiwifruit orchards and wheat-maize rotation farmland. The variation coefficient of all soil nutrients belonged to moderate spatial variability, mainly associated with different management and fertilization status. Yu et al. [20] based on the regional field investigation and laboratory analysis, studied the large-scale spatial heterogeneity and distribution pattern of limestone soil nutrients in the karst area in northwestern Guangxi by combining classical statistics and geostatistics. The results showed that the pH value of soil in karst area did not change much, while the variation coefficient of soil nutrients varied from 30% to 75%. The spatial variability of soil nutrients is different. Spherical model, gaussian model, and linear model are the best fitting models for SOM, AP, and available potassium (AK). However, pH, available nitrogen (AN), TN, total phosphorus (TP), and total potassium (TK) can be most appropriate using the exponential model. Zhang et al. [21] based on Jiangxi province 340 forest soil profiles soil nutrient data, using statistical methods and GIS technology to study the spatial variation characteristics of soil SOM, TN, and TP. The results showed that the spherical model, exponential model, and gaussian model can describe the spatial variation of soil nutrients. Dai [22] based on the data of the 45 profiles of the second national soil survey in 1985 and the 61 profile of the soil survey in 2015 in the Huangshui River Basin of Qinghai Province, used the classical statistics including descriptive statistics analysis, correlation analysis, and variance analysis, and the geostatistics including semi-variance analysis and ordinary kriging analysis, for spatio-temporal variations of TN and their influencing factors in the past 30 years. Results indicates that soil TN content during the period varied at a medium level; semi-variance analysis shows that the TN data in 1985 fit the exponential model, while those in 2005 fit the Gaussian model and the spherical model, respectively.

The variability of the soil nutrients is not only manifested spatially but also temporally. The factors that cause changes in the spatiotemporal variation of soil nutrients are complicated, including not only natural factors (such as parent materials, climate, and topography) but also human factors (such as fertilization, land use modes, and management levels). At present, long-term excessive fertilization has caused problems such as unbalanced soil N and P contents and low utilization efficient of chemical fertilizers. Study of the spatiotemporal variations in soil nutrients and exploration of the influence of human activity factors on soil environment have important implications for precision agriculture, protection of land resources, and protection of farmland ecological environments.

At present, the spatiotemporal variability of soil nutrients and soil properties have been studied in-depth in China and abroad, but few studies exist on the spatiotemporal variability at a large scale. In addition, the interpolation precision can be a problem in some aspects, for example, the selection of fitting curve of semivariance function, model selection in statistics and the effects of the sample number. In this study, best-fitting models for interpolation analysis were selected based on the location and properties of the sampling points.

The best-fitting interpolation model was selected for SOM, N, P, pH, and electric conductivity (EC), and ordinary kriging interpolation was performed to analyze the soil spatiotemporal variability within the study region for each of these factors. Specifically, the selection of best-fitting models and spatial interpolation parameters were conducted by the GS + Statistics method, and the kriging spatial interpolation was conducted by the ArcGIS software (10.2, ESRI, Redlands, CA, USA). This study mainly includes the selection of best-fitting models and spatial interpolation parameters, statistical characteristic analysis of soil nutrients in the dry season and rainy season, spatial variation analysis of soil nutrients, and spatial pattern analysis of soil nutrients.

## 2. Materials and Methods

### 2.1. General Information of Study Area

**Figure 1:**
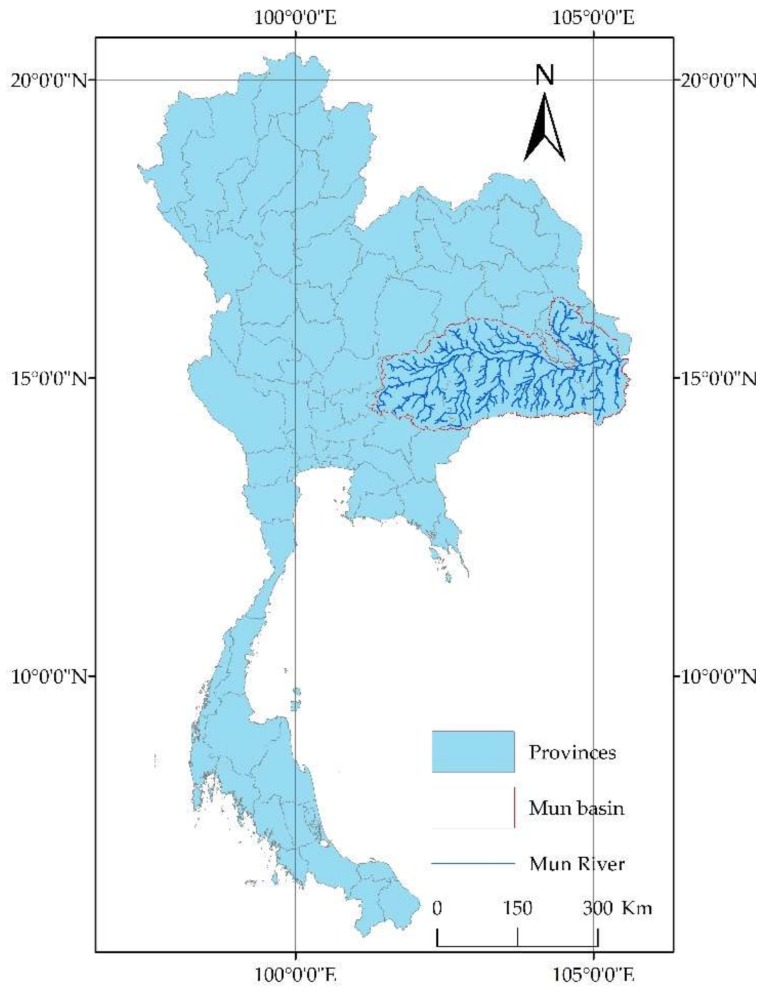
Location of Mun River Basin.

The Mun River (Figure 1) is approximately 673 km long and has a river basin acreage of approximately 82,000 km^2^. Its upstream is located in the Korat Plateau and flows through 10 provinces and finally enters into the Mekong River at the junction of Thailand and Laos. Within the basin, the terrain is high in the west and low in the east; plateaus and mountains are in the southwestern area, and plains are in the central and eastern areas. In the southwestern highlands, mountains and rivers are alternately distributed, with most of them running in a south–north direction in a vertically distributed manner. In the central and eastern regions, many plains and rivers run in the south–north direction, which provides sufficient water resources for the water cycling in these plain regions.

The Mun River Basin has a humid subtropical climate, and the climate is most significantly affected by the tropical monsoons in Asia. The climate and hydrology within the basin show significant seasonal differences [23]. Changes brought with the seasonal monsoon render distinct wet and dry seasons in the basin. The annual temperature is not lower than 18 °C, and the average annual rainfall is 1300–1500 mm.

In the summer, the southwest monsoon blowing from the Indian Ocean generates high temperatures and abundant rainfall and it is generally referred to as the ‘rainy season’. The rainy season lasts from mid-May to the beginning of October, with heavy rainfall generally being concentrated in August or September. In winter, due to the strong Mongolian cold and high pressure, the northeast monsoon brings low temperatures and dry weather, and this period is generally referred to as the ‘dry season’. The dry season lasts from November to April of the following year, with an average temperature of 16 °C (high temperature can be up to 40–42 °C). The transitional season is between the dry season and the rainy season, and frequent non-directional winds occur at this time [24]. With the dry season and rainy season resulting from the monsoon effect, an extremely uneven distribution of rainfall exists during the year.

### 2.2. Sample Collection

**Figure 2:**
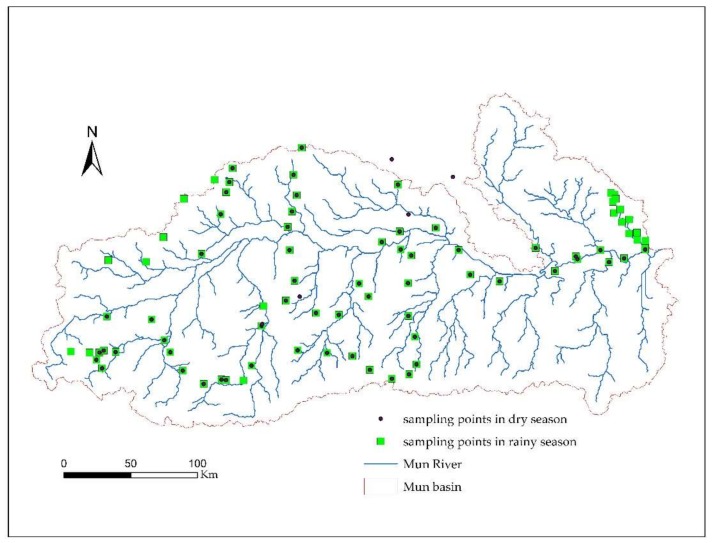
Distribution map of the soil sampling points.

The typical sample plot method was used regularly by taking into account different soil types and land use patterns. The sampling time was in February 2017 (for the dry season) and in August 2017 (for the rainy season). Soil samples in the profile were taken from representative areas and treated by the quaternary method upon even mixing, and the remaining 1.5 kg samples were brought back to the laboratory for air drying. After roots, leaves, and stones were removed, samples were stored for further use. To ensure the consistency of the sampling habitat, the slopes of the sampling quadrats were all <5°. The latitude and longitude coordinates of each sample were recorded with GPS, and at the same time, vegetation growth and land use conditions and soil moisture were measured and photographed around the sample sites. Because only data at shallow soil layers were used in this study, the soil sampling depth was set as 0–20 cm. A total of 66 samples in the dry season and 86 samples in the rainy season were included, with the distribution of specific sampling points shown in Figure 2.

### 2.3. Sample Analysis Methods

Available phosphorous (AP) was determined by using the 0.50 mol/L sodium bicarbonate extraction–molybdenum antimony anti-colorimetric method. The TN content was determined by the Kjeldahl method. The SOM was measured by the dry combustion method, which is a method that is not only complicated and time-consuming to perform but also requires advanced analytical techniques and special instruments and is expensive [25]. Soil EC was measured using the EC meter. The soil pH value was measured using the potentiometric method. Soil texture was determined using the hydrometer method [26,27].

### 2.4. Data Processing

#### 2.4.1. Selection of Best-Fitting Model

Based on the spatial variability of the soil nutrients revealed by geostatistics, the nugget and sill values were calculated, in turn, providing parameters for the kriging interpolation.

First, the semivariance function of samples was calculated using the formula

(1) $$\gamma (h)\;=\;\frac{1}{2N\left(h\right)}\overset{N\left(h\right)}{\underset{i=1}{\sum }}{\left[Z\left(x_{i}+h\right)-Z\left(x_{i}\right)\right]}^{2}$$

where γ(h) is the semivariance of the samples, h is the distance between two sampling points (also referred to as the lag distance), N(h) is the number of paired data at a distance of the interval h, and $Z\left(x_{i}+h\right)\;\mathrm{and}\;Z\left(x_{i}\right)$ are the measured values at the sampling point of $x_{i}+h$ and of $x_{i}$, respectively. The semivariance scatter plots calculated from the actual sampling points need to be fitted using the semivariance model to obtain the spatially related semivariance curves. In this study, the gaussian model, spherical model, exponential model and linear model were used to fit the optimal model [28].

The selection of the optimal fitting model is determined by the sum of maximum decision coefficient and minimum residual squares.

The descriptive statistical analysis for soil nutrients was performed with the SPSS software (22.0, IBM, Armonk, NY, USA), and the K-S test was used to perform nonparametric tests, in which the significance levels set as 0.01 and 0.05. Spatial autocorrelation analysis, semivariance function analysis, optimization, selection of models and simulations, and calculation of parameters, as well as the selection of best-fitting models, were all performed with GS + Statistics software (7.0, Gamma Design Software, LLC, Plainwell, MI, USA), which in turn provided parameters for spatial interpolation. The kriging spatial interpolation was performed using the ArcGIS software.

#### 2.4.2. Methods for Validation and Evaluation

The leave-one-out cross-validation test was used to evaluate the interpolation effect. Mean standard error (MSE), bias, precision, relative accuracy, and root mean square error (RMSE) were used to evaluate the interpolation effect.

Using the interpolation parameters provided by the best-fitting model, precision testing of the interpolation results was performed with the ordinary kriging method to validate the feasibility of the study method.

**Figure 3:**
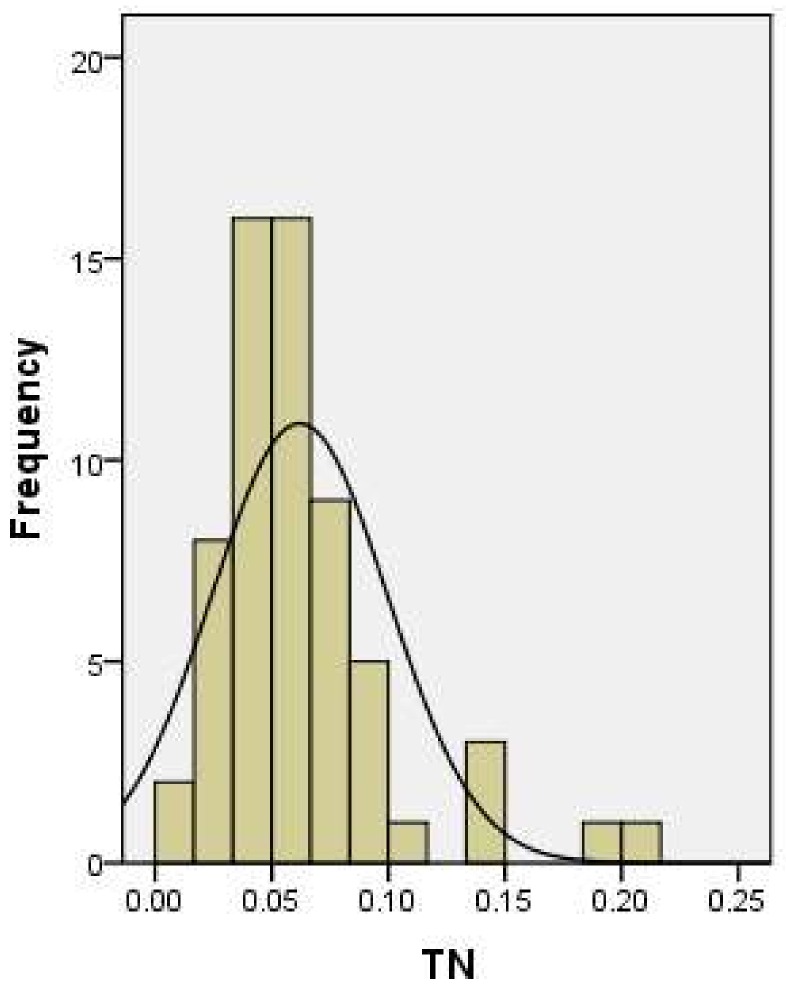
Histogram of soil TN content in sample site.

It can be seen from the histogram of Figure 3 that the observation points follow a normal distribution, and no outliers. The measured value of TN was tested by K-S, and the progressive significance value was 0.112, greater than 0.05, indicating that the experimental sample can be used for further analysis.

**Figure 4:**
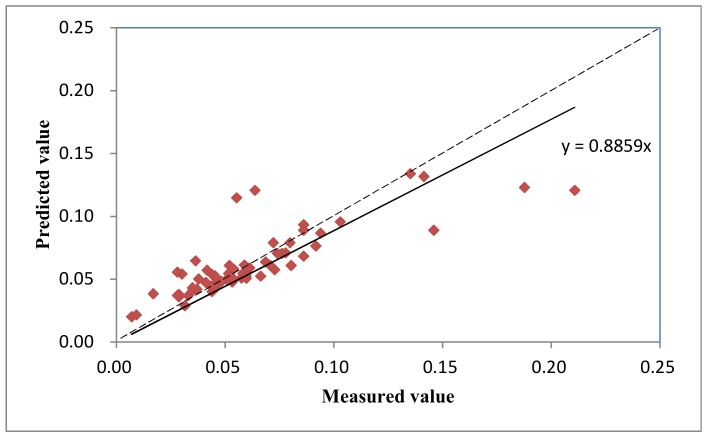
Cross-validation test of ordinary kriging interpolation for soil TN content (dashed line denotes the 1:1 line).

The ordinary kriging interpolation method was used to perform the best fitting unbiased spatial interpolation for spatially unsampled points, and the leave-one-out method was used for the cross-validation test. Figure 4 shows the relation between the measured values and predicted values of soil-TN in the dry season for the sampling points, and the paired data points with centered soil TN content values are distributed closely around the 1:1 reference line, which indicates that the predicted values in this range are very close to the measured values, showing a high precision of prediction. The overall predicted values tend to approach the mean value and showed a pattern that the sampling points with relatively low content are overestimated, whereas the sampling points with relatively high content are underestimated. Such a pattern is the well-known kriging interpolation smoothing effect [29], and this effect is a common problem of weighted average-based interpolation methods. The regression coefficient between the predicted and measured values is close to 1, indicating that kriging interpolation precision is high, and the spatial distribution map obtained by using this method can accurately reflect the actual situation.

## 3. Results

### 3.1. Classical Descriptive Statistical Analysis

The distribution characteristics of soil nutrients in the Mun River Basin were analyzed (Table 1) using classical statistical parametric methods; the differences in the soil nutrients between the dry season and rainy season were determined, and the temporal variation of each soil nutrient was studied.

As revealed by the data in the sampling points, soil pH was mildly acidic, and the EC and AP content showed a relatively large seasonal difference whereas the SOM and TN contents were relatively stable: soil acidity and TN content in the rainy season > soil acidity and TN content in the dry season, soil EC, SOM and AP in the dry season > soil EC, SOM, and AP in the rainy season. Specifically, pH and SOM showed small variation, 12% and 62% in the dry season and 17% and 66% in the rainy season; EC and AP showed large variation, 92% and 113% in the dry season and 133% and 89% in the rainy season; and TN remained basically stable, with the coefficient of variation (CV) being 60% in the dry season and 51% in the rainy season.

The correlation analysis of the soil elements in the dry season is shown in Table 2. The soil pH value was significantly correlated with elevation and EC, soil EC was significantly correlated with SOM and TN content, SOM content was significantly correlated with soil texture, AP, and TN, soil AP content was significantly correlated with SOM and TN content, and soil TN content was significantly correlated with soil texture, SOM, and AP content.

The correlation analysis of the soil elements in the rainy season is shown in Table 3. The soil pH value was significantly correlated with elevation and EC, soil EC was significantly correlated with soil texture and SOM, soil EC also had a significant correlation with TN content, SOM was significantly correlated with soil texture and TN content.

### 3.2. Spatial Variability Analysis

The parameters provided by the geostatistical analysis further revealed the spatial variation characteristics of the soil nutrients, pH, and EC at the regional scale in the Mun River Basin. Specifically, nugget (C_0_) refers to random variations that may be caused by human activities, soil biology, and sampling errors, whereas sill (C_0_ + C) represents the total variation in the soil nutrient content at the current scale.

**Figure 5:**
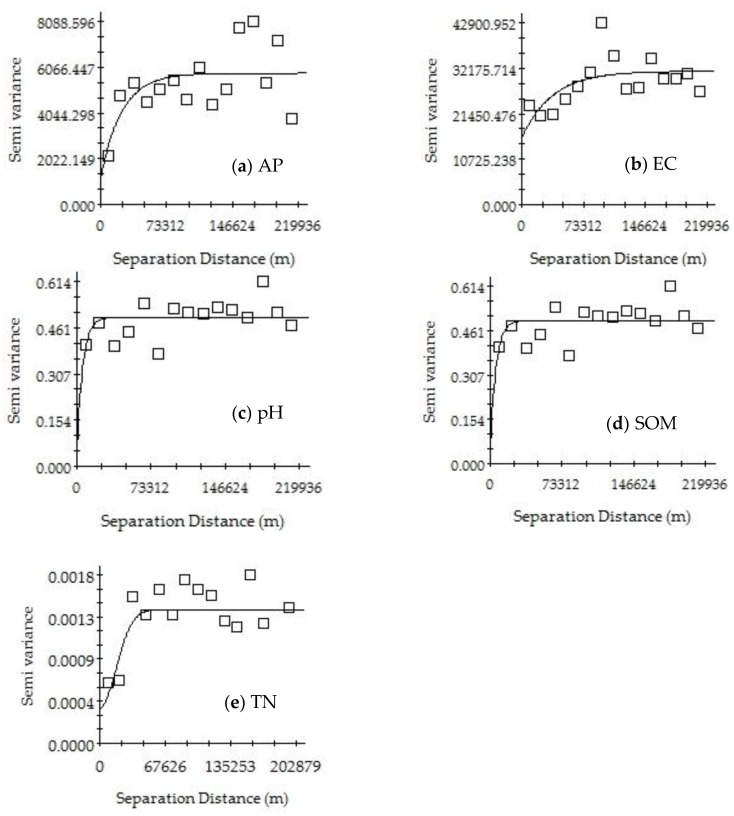
Variograms of AP (**a**), EC (**b**), pH (**c**), SOM (**d**) and TN (**e**) in the dry season of Mun River Basin.

**Figure 6:**
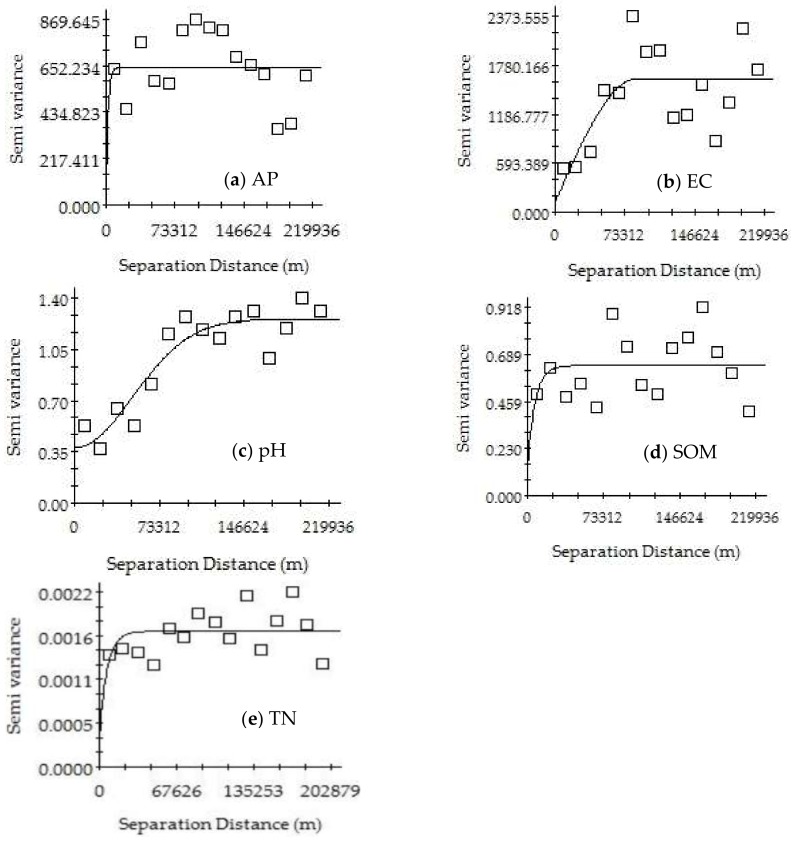
Variograms of AP (**a**), EC (**b**), pH (**c**), SOM (**d**) and TN (**e**) in the rainy season of Mun River Basin.

Table 4, Figure 5 and Figure 6 show the results of the geostatistical analysis of the SOM, TN, AP, EC, and pH in the Mun River Basin. The results show that for the same indicator, the best-fitting model may be different or may be the same in the dry season versus the rainy season. The best-fitting model was selected for further analysis. The spatial structure characteristics of soil were relatively consistent with the planting system in the study area. Such consistency in the planting system made the soil nutrients basically stable over time, and the spatial distribution showed an adequately regular pattern.

The soil in the study area had a good spatial structure and showed an adequate degree of spatial autocorrelation. The best-fitting model for the EC in the dry season was the Gaussian model, and it had a moderate spatial autocorrelation; the best-fitting model for the EC in the rainy season was the spherical model, in which the nugget effect value was greater than 0.75, indicating that the system spatial correlation was very weak and that the spatial variability of the samples was caused by random factors.

The best-fitting model for pH was the exponential model in the dry season and Gaussian model in the rainy season. The nugget effect value in the dry season was greater than 0.75, indicating a very weak system spatial correlation. The nugget effect value in the rainy season had a range of 0.25–0.75, showing moderate spatial autocorrelation.

The best-fitting model for TN in the dry season was the Gaussian model, with a nugget effect value of 0.71 and showing a moderate spatial autocorrelation; the best-fitting model for TN in the rainy season was the exponential model, in which the nugget effect value was greater than 0.75, indicating a very weak spatial correlation for the system.

The best-fitting model for AP in the dry and rainy season was the exponential model. The nugget effect value in the dry season had a range of 0.25–0.75, showing a moderate spatial autocorrelation; the nugget effect value in the rainy season was greater than 0.75, indicating a very weak system spatial correlation.

The best-fitting model for SOM in the dry season and the rainy season was the exponential model, and the nugget effect values in both seasons were greater than 0.75, indicating a very weak system spatial correlation.

### 3.3. Spatial Pattern Analysis

#### 3.3.1. Distribution Characteristics in Dry Season

**Figure 7:**
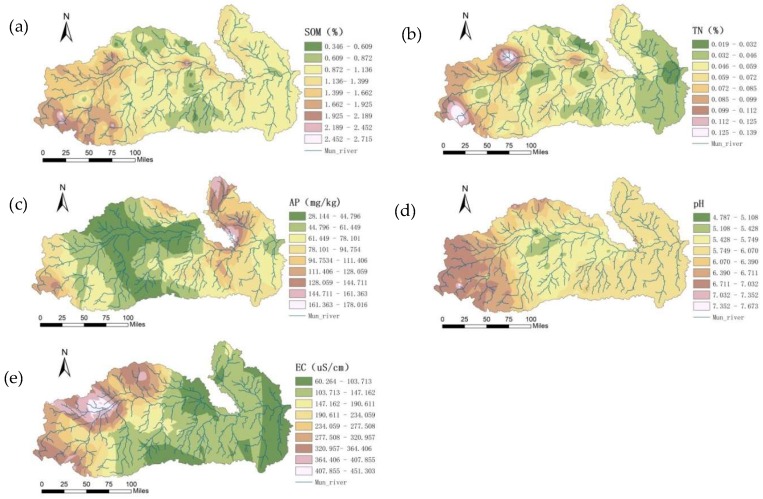
Spatial patterns of SOM (**a**), TN (**b**), AP (**c**), pH (**d**), and EC (**e**) in the dry season.

Based on the obtained semivariance function model and related parameters, the ordinary kriging method was used to perform optimal spatial interpolation for SOM, TN, AP, pH, and EC in dry season, and the spatial distributions of SOM, TN, AP, pH, and EC were graphed (Figure 7), providing an intuitive description of the spatial distribution of the soil characteristics.

The spatial distributions of SOM were affected by various systems or random factors and showed complex spatial variability; yet, the interpolation results could still explain the pattern of the SOM distribution. The SOM showed a concave distribution as a whole, with a patchy distribution in some areas. The central plains had a flat terrain and were mostly farmland, and the SOM content was the highest; the area of the upper reaches had a relatively high terrain and was mostly dry land and forest land, and the SOM content was high; the lower reaches had the lowest SOM content.

The TN content was significantly correlated with the SOM, AP, and soil sand content at the 0.05 level. The spatial distribution of the soil TN content was similar to that of SOM, showing that the spatial correlation of TN and SOM was strong. The soil TN contents were in the medium to high levels in some regions of the upper and lower reaches, whereas it was low in the middle reaches.

The soil AP content was low in the middle reaches and was at the medium and high levels in the upper and lower reaches. In the middle reaches, the irrigation conditions were good with a sufficient water supply and with two to three crop growth seasons per year; the soil was subjected to great interference by human activities, and crop uptake and flooding had a greater impact on AP. The spatial distribution of AP was mainly related to fertilization, cycling, and mobility of the phosphorus.

The pH was relatively low in the middle reaches, which was related to the leaching of calcium and magnesium ions due to the high rainfall in the central region. In addition, the high SOM also produced organic acids and lowered the pH values. The upper reaches had high pH values, with the sandy soil, the evaporation amount was greater than the precipitation, which resulted in serious soil salinization. The irrigation resulted in the accumulation of salt in the soil and increased the pH value. pH values in the lower reaches were relatively high.

The EC was high in the upper reaches and low in lower reaches, which was closely related to land use/planting patterns, geographical factors, and climatic factors. The upper reaches were mostly mountainous, and its land type was dominated by forest land; during the dry season, its soil had much moisture with many free ions, which naturally rendered high EC values. The lower reaches were mostly plains, which were fallow during the dry season; the soil surface of these plains was bare and exposed to sunlight, and the water content was low, with few free ions, which naturally rendered low EC values.

#### 3.3.2. Distribution Characteristics in Rainy Seasons

**Figure 8:**
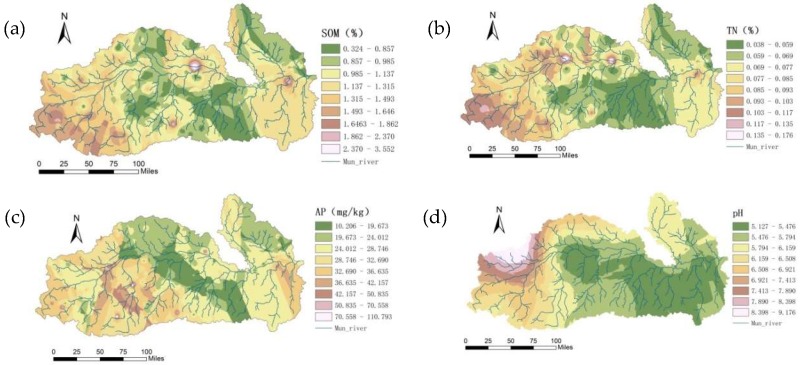

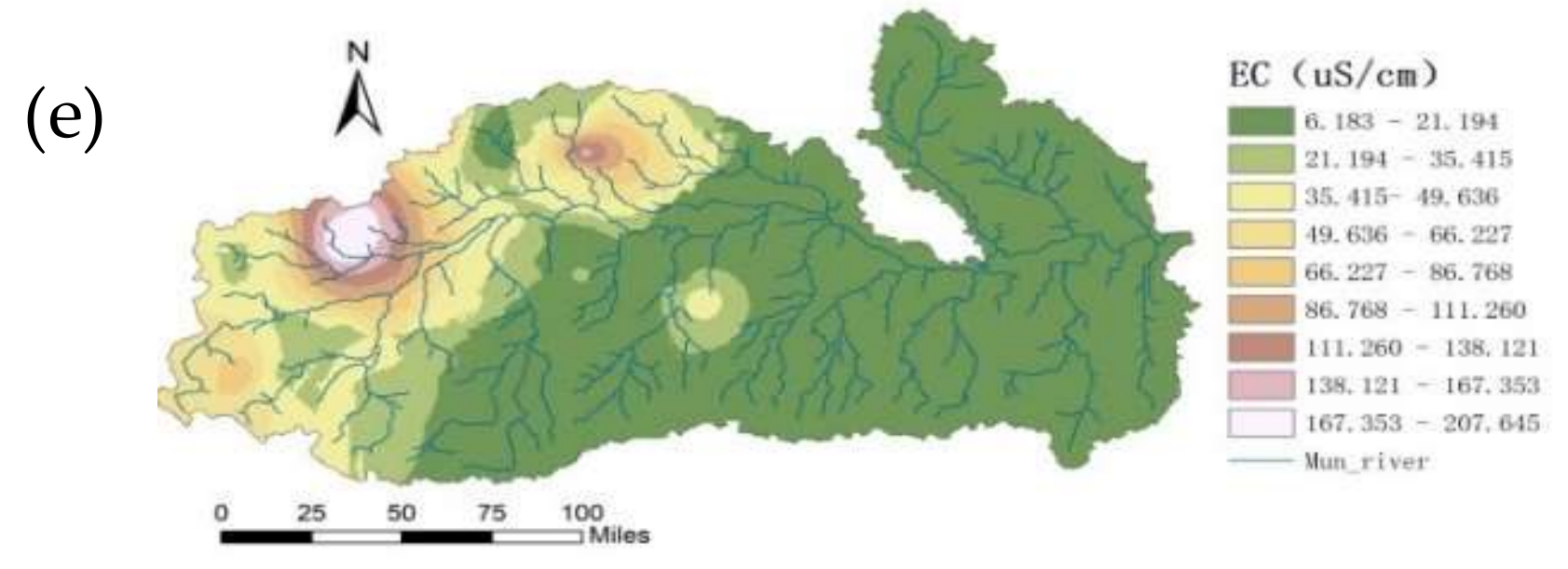
Spatial patterns of SOM (**a**), TN (**b**), AP (**c**), pH (**d**), and EC (**e**) in the rainy season.

Ordinary kriging was used to perform optimal spatial interpolation on the SOM, TN, AP, pH, and EC based on the obtained semivariance function model and related parameters, and the spatial distributions of SOM, TN, AP, pH, and EC were graphed (Figure 8) to intuitively describe the spatial distribution of the soil properties in the study area.

The spatial pattern of SOM was similar to that in the dry season, and SOM was relatively stable within a year. The SOM content in the upper and lower reaches was at medium and high levels, whereas that in the middle reaches of the region was at a low level.

The spatial distribution of soil TN was similar to that of SOM, indicating that the spatial correlation between TN and SOM was strong. The TN content was in the middle to high levels in the upper and lower reaches and was relatively low in the middle reaches.

The spatial pattern of AP was similar to that in the dry season, with both seasons showing a relatively low pattern in the central region yet a high value in the upper and lower reaches. During the rainy season, with the growth of crops and with sufficient water flow in the rivers, AP was, on one hand, taken up and utilized by crops and, on the other hand, was flowing into rivers along with the surface water, causing water pollution. The spatial distribution of AP in the rainy season had a close relation with fertilization, uptake, and utilization by crops and mutual flow of surface water and river water.

The spatial distribution of pH was generally similar to that in the dry season, albeit with differences in some localities. The pH value in the middle was low, and high in the upper reaches, followed by the lower reaches.

In the rainy season, the spatial pattern of EC was similar to that observed in the dry season, showing a trend of high in the upper reaches and low in the lower reaches. EC is influenced by factors such as salt content, moisture, temperature, SOM content, and soil texture. Soil EC is an important parameter for studying agriculture because it reflects the quality and physical properties of soil, and it is an important guide for reasonable fertilization and irrigation.

#### 3.3.3. Comparative Analysis of Soil Nutrients in the Dry Season and Rainy Season

**Figure 9:**
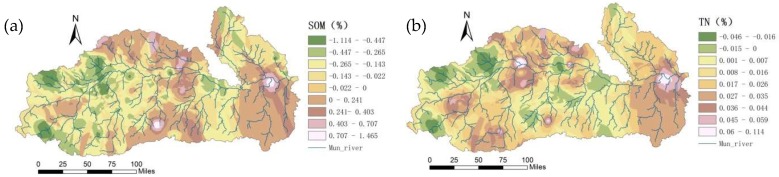

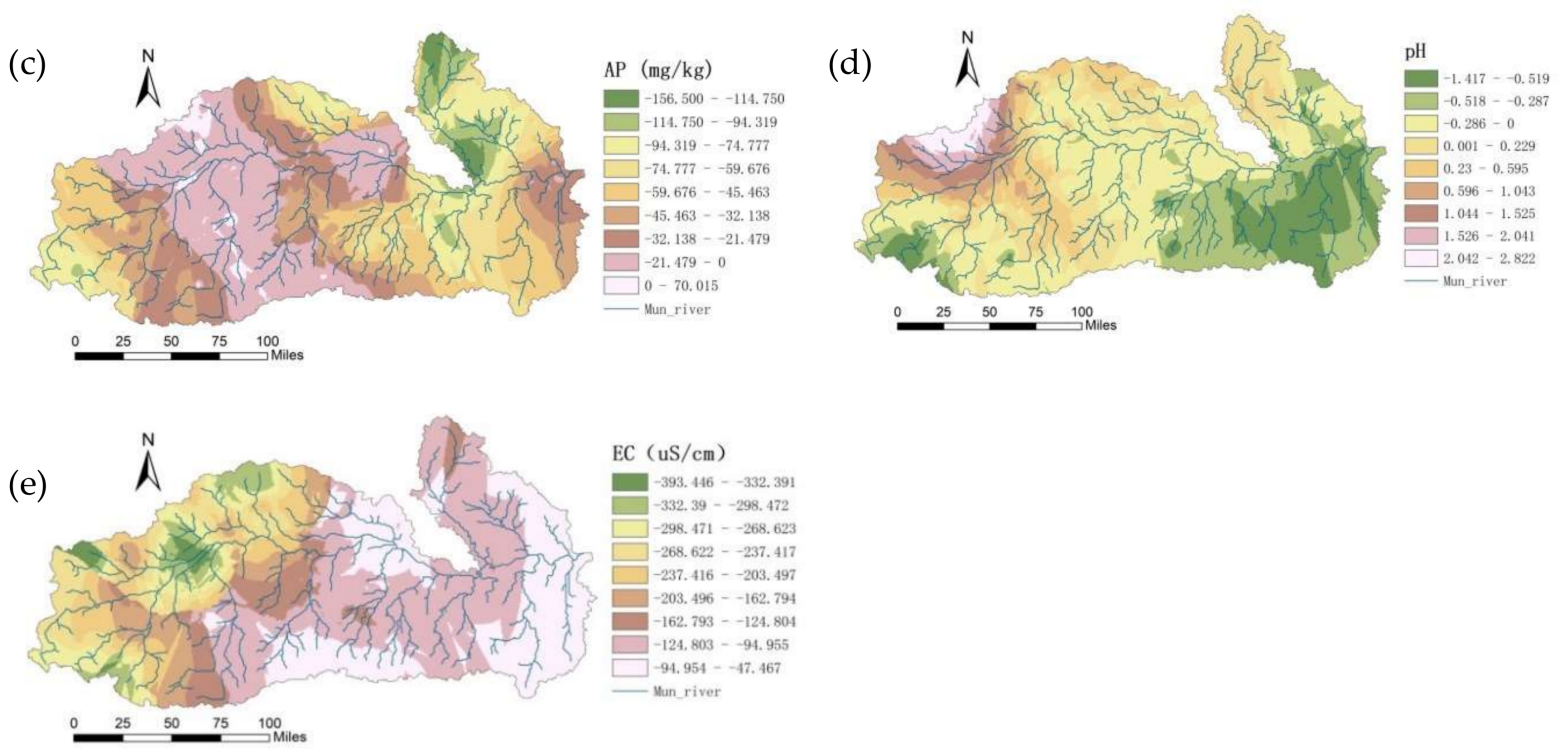
Spatial differences of SOM (**a**), TN (**b**), AP (**c**), pH (**d**), and EC (**e**) between the dry and the rainy season.

**Figure 10:**
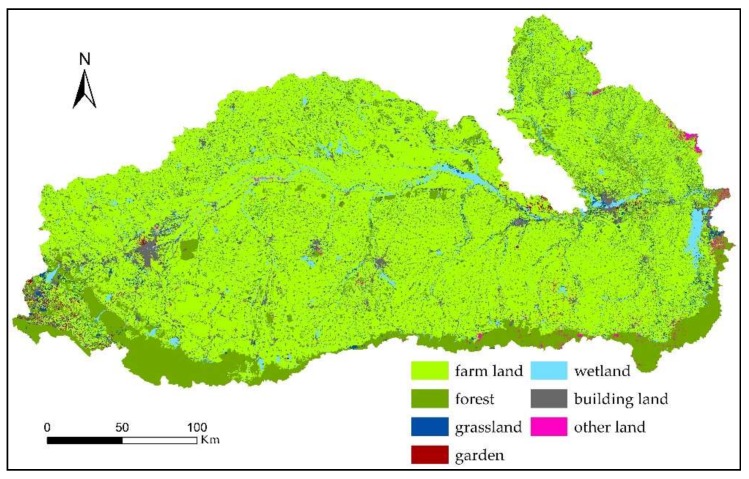
Landuse map of the Mun River Basin.

According to the spatial patterns of the soil nutrients in the dry season and in the rainy season in the Mun River Basin, changes in the spatial patterns of soil nutrients (Figure 9) were analyzed by using the GIS statistical analysis function. The differences between the rainy season and the dry season for elements such as soil N and P and SOM were analyzed, and the relation between these differences and land use (Figure 10) and environmental factors was explored.

SOM generally showed a decreasing trend. The SOM contents in most areas of the upper and middle reaches in the rainy season were lower than those in the dry season; most of these areas were paddy field. The SOM contents in the edge area of the middle reaches and in some areas in the lower reaches in the rainy season were higher than those in the dry season; most of these areas were forest and non-irrigated farmland.

In the upper reaches, the elevation is relatively high, and the vegetation type is mainly forestland. The agricultural land had mostly a one-crop growth season per year, which had less human interference than the middle reaches, reducing the chance of SOM exposure to the air and thus the decomposition of the SOM; over the long term, the SOM accumulation was accelerated. Hence, the SOM in the upper reaches generally exhibited medium to high levels. In addition, the soil in the middle reaches was mostly sandy, with a low ability for SOM sequestration, leading to a slow rate of SOM degradation.

The areas with lower elevations in the middle reaches are mostly plain areas and river valleys. These areas had a large acreage of agricultural land, and the content of SOM was thus greatly affected by human activities. On the one hand, because the middle reaches were irrigated, agricultural measures such as irrigation and fertilization promoted the growth of crops and increased SOM. On the other hand, frequent floods in recent years have scoured the surface soil, which not only resulted in the loss of surface soil and destroyed the soil structure but also reduced SOM input. In addition, the impact of human planting activities (such as soil tillage, soil turning, and fallowing) increased SOM exposure to the air and accelerated SOM degradation. SOM was in a dynamic state between input and output, resulting in a low SOM content in the middle reaches. The long-term immersion of river water was conducive to the accumulation of SOM, and the SOM content around the river trunk was at a medium to high level.

The overall trend of soil TN showed an increasing trend, with great seasonal variation. Because the rainy season was the farming season, a large acreage of the basin was farmland, and the influence of fertilization led to an increase in N. The rainy season had plenty of rainfall, and N was transferred from the soil into the water due to leaching, which in turn resulted in N moving along with water flow to cause an increase in N in the lower reaches. The distribution of TN had a close relation with the land use mode. Under the joint action of human activity-induced environmental and geographical factors, TN showed obvious seasonal differences. The soil TN content in the lower reaches in the rainy season was significantly higher than that in the dry season, followed by soil TN content in the middle reaches. The soil TN content in some sporadic areas in the upper reaches in the rainy season was lower than that in the dry season, showing a decreasing trend.

According to the spatial pattern of AP content in the dry and rainy seasons, the AP in the entire basin in the rainy season was lower than that in the dry season, showing a significant decreasing trend. Rainy seasons are supposed to have high surface P due to human activities, but the study area showed the opposite trend: from the dry season to the rainy season, only the AP content of some sporadic lands in the middle reaches of the irrigated area was increased, whereas the remaining areas showed a decreasing trend. Specifically, AP content in the northeastern part of the upper reaches decreased by 155–115 mg/kg, whereas AP in the central irrigated area was rather stable, decreasing by 21 mg/kg on average. AP content was decreased from the middle reaches to the lower and to the upper reaches, especially with a significant decreasing trend between the middle reaches and the lower reaches, showed a close relation with the topographical factors, the direction of the river flow, and the type of planted crops.

Soil P had a very large seasonal variation, and AP content in the dry season was significantly higher than that in the rainy season. First, this finding was related to the local nutrient management measures of valuing N over P. Based on the analysis of 61 effective household survey, the main chemical fertilizers in farmlands were inorganic fertilizers (N-P-K), mainly including application schemes of 16-8-8, 15-15-15, 46-0-0, 27-12-6, 16-20-0, 16-16-8, and 18-4-5. According to the N, P, and K contents and the total application rate of fertilizers, N accounted for 53.33%, AP accounted for 25.94%, and K accounted for 20.72%; Second, the rainfall in the rainy season was relatively large, and the nutrient loss associated with the water was also the main reason for the reduction in soil AP. Finally, the soil parent material, topography, and land use patterns also had an impact.

The pH changes showed a gradual decreasing trend from the northwest to the southeast. The change in pH value can directly or indirectly affect the nitrification and denitrification in the soil, and the change in the trend of the pH value is generally similar to that of the TN content. The change in the soil pH value is influenced by human activities such as fertilization and irrigation; and is also related to factors such as topography, elevation, soil moisture, and rainfall.

From the dry season to the rainy season, the EC was gradually increased from the upper reaches to the lower reaches, with an overall decreasing trend. Soil EC was decreased by 393–332 µS/cm from the dry season to the rainy season in some of the western regions and was decreased by 95–47 µS/cm in the eastern region. Changes in EC are mainly affected by soil moisture, temperature, and the number of ions in the soil solution. In the rainy season, the water content was large, the ion flow rate was fast, and the ion concentration in the soil solution was reduced, which resulted in a significantly lower EC in the rainy season. This change resembled the change in soil nutrients, which showed a gradual increase from the upper reaches to the lower reaches; this also provided mutual evidence of the poor water quality in the lower reaches of the basin.

## 4. Discussion

The weathering of minerals under natural conditions is the main source of soil nutrients, but the degree and process of human activities under different land use patterns are also important influences on soil nutrients. Different land use patterns change the soil microecological environment so that the changes in soil nutrient content show different trends.

SOM, TN, AP, pH, and EC in the Mun River Basin showed significant spatial variations. According to the analysis of the differences between the dry season and the rainy season, the output potential of nonpoint source pollutants in the soil was large, and the land use pattern and fertilizer use amount had significant effects on soil nutrient content.

SOM is an important aspect of soil fertility, and its content is significantly impacted by land use patterns. In this study, the changes in the spatial pattern of SOM showed a significant correlation with land-use patterns, and the SOM content exhibited a pattern of forestland > dryland > paddy field. The nature of such a spatial pattern for SOM indicated that the land use pattern in this region had a significant impact on SOM content. Under different land use patterns, the SOM content of forestlands is significantly higher than that of cultivated land or land used for other reasons. Subjected to long-term natural conditions, forestlands experience little human disturbance, which causes the SOM input to be greater than the output; additionally, litter, plant roots, and humus are rapidly degraded under the action of soil microorganisms; and all these are the main factors for high SOM content in forest lands. In contrast, cultivated land and other farmland are subjected to frequent cultivation, field management measures, and fertilization methods, which result in the destruction of soil structure and in the exposure of SOM in the granule structure to the air, thereby accelerating the decomposition of SOM.

The spatial pattern of soil pH has obvious consistency with the land use pattern. The forestland had a low pH and exhibited strong soil acidity: the organic acids and enzymes released to the soil from the plant litters and roots, the shed root cap cells, and the residual roots not only increase the SOM content but also contribute to the activity of the microbes, facilitating the dissolution of plant nutrients and increasing the soil fertility; organic acids also neutralize OH- ions in the soil, thereby reducing soil pH. Although soil pH value does not directly indicate the content of a certain nutrient in the soil, its level can control and influence the change of microflora in the soil, thus affecting most elements’ conversion direction and process as well as their states and their availability.

In addition, the selection of the correct sampling points and the correct analysis of the collected data are the most important steps in the research. The evaluation quality depends on the accuracy by which each of these phases has been performed. However, in the study, the number of measuring points was scarce as the Mun River Basin is such vast area. Therefore, the geostatistical analyses as kriging can only produce rough estimations. More precise research with more samples needs to be done in the next step.

## 5. Conclusions

Changes in land use patterns can cause changes in many natural phenomena and ecological processes such as soil nutrients, soil moisture, soil erosion, biodiversity, and biogeochemical cycles. Soil nutrients are an important component of soil fertility and have an important influence on the structure and function of ecosystems. Meanwhile, land use has an important influence on soil quality and that land with a long time as farmland return is of good soil quality. According to this study, the influence of land use patterns in the Mun River Basin on soil nutrients had a significant seasonality, and geographic factors also influenced the distribution of soil nutrients. This study showed that soil acidity was higher in the dry season than in the rainy season, soil EC was significantly higher in the dry season than in the rainy season, SOM and AP contents were higher in the rainy season than in the dry season, and soil TN content was higher in the rainy season than in the dry season. The CV values for pH, EC, and SOM in the rainy season were greater than those in the dry season, and the CV values for the AP and TN content in the dry season were greater than those in the rainy season. Due to the interference from human activities, the increase in cultivation intensity had contributed to potential nonpoint source pollution in the basin. This study also found that the eutrophication of river water bodies was serious and that the salinization degree was relatively high in some regions of the study area.

## Figures and Tables

**Table 1:** Statistical characteristics of soil nutrient, pH, and conductivity at sampling sites.

Item	Time	Mean	Median	STD	CV %	Kurtosis	Skewness	Region	Minimum	Maximum
pH	Dry season	6.03	5.80	0.71	0.12	0.03	0.79	3.40	4.60	8.00
	Rainy season	6.11	5.76	1.07	0.17	2.72	1.64	5.05	4.69	9.74
EC	Dry season	183.02	119.84	168.94	0.92	2.39	1.70	710.33	21.67	732.00
	Rainy season	28.87	16.03	38.52	1.33	13.71	3.52	223.89	4.11	228.00
SOM	Dry season	1.23	1.06	0.76	0.62	1.24	1.17	3.46	0.10	3.56
	Rainy season	1.16	0.95	0.76	0.66	9.95	2.33	5.31	0.05	5.36
AP	Dry season	63.45	39.22	71.93	1.13	12.39	3.45	382.44	22.80	405.23
	Rainy season	28.18	19.84	25.17	0.89	7.96	2.68	132.59	6.88	139.47
TN	Dry season	0.06	0.05	0.04	0.60	5.29	2.01	0.20	0.01	0.21
	Rainy season	0.08	0.07	0.04	0.51	3.79	1.71	0.21	0.03	0.24

**Table 2:** Pearson coefficient of soil nutrient and elevation and soil texture in dry season.

Item		Ele	PH	EC	Silt + Clay	Clay	Sand	Silt	SOM	AP	TN
Ele	Pearson	1	0.528 **	0.168	0.165	0.273 *	−0.165	−0.141	0.310 *	0.029	0.388 **
	Significance		0.000	0.178	0.186	0.026	0.186	0.259	0.011	0.818	0.001
pH	Pearson		1	0.273 *	0.063	0.158	−0.063	−0.166	0.164	0.218	0.206
	Significance			0.026	0.614	0.204	0.614	0.183	0.188	0.078	0.098
EC	Pearson			1	0.197	0.223	−0.197	0.049	0.389 **	0.230	0.451 **
	Significance				0.112	0.071	0.112	0.697	0.001	0.063	0.000
Silt + Clay	Pearson				1	0.934 **	−1.000 **	0.662 **	0.645 **	−0.005	0.526 **
	Significance					0.000	0.000	0.000	0.000	0.966	0.000
Clay	Pearson					1	−0.934 **	0.350 **	0.672 **	0.016	0.551 **
	Significance						0.000	0.004	0.000	0.896	0.000
Sand	Pearson						1	−0.662 **	−0.645 **	0.005	−0.526 **
	Significance							0.000	0.000	0.966	0.000
Silt	Pearson							1	0.282 *	−0.048	0.223
	Significance								0.022	0.700	0.072
SOM	Pearson								1	0.360 **	0.869 **
	Significance									0.003	0.000
AP	Pearson									1	0.513 **
	Significance										0.000
TN	Pearson										1
	Significance										

** Significant correlation at layer 0.01 (double-tailed); * The correlation was significant at 0.05 layers (double-tailed); ‘SOM’ represents soil organic carbon concentration; ‘Clay’ represents the content of soil clay particles; ‘Silt’ represents the content of soil particles; ‘Ele’ represents the altitude; ‘pH’ represents pH of soil.

**Table 3:** Pearson coefficient of soil nutrient and elevation and soil texture in rainy season.

Item		Ele	PH	EC	Silt + Clay	Clay	Sand	Silt	SOM	AP	TN
Ele	Pearson	11	0.218 *	0.207	0.355 **	0.376 **	−0.352 **	0.129	0.179	0.132	0.240 *
	Significance		0.044	0.055	0.001	0.000	0.001	0.236	0.099	0.226	0.026
pH	Pearson		1	0.537 **	0.180	0.201	−0.178	0.039	0.157	0.042	0.174
	Significance			0.000	0.098	0.064	0.100	0.723	0.148	0.704	0.110
EC	Pearson			1	0.313 **	0.294 **	−0.310 **	0.207	0.221 *	0.034	0.282 **
	Significance				0.003	0.006	0.004	0.056	0.041	0.754	0.009
Silt + Clay	Pearson				1	0.951 **	−0.999 **	0.635 **	0.530 **	0.011	0.567 **
	Significance					0.000	0.000	0.000	0.000	0.920	0.000
Clay	Pearson					1	−0.951 **	0.366 **	0.557 **	0.029	0.578 **
	Significance						0.000	0.001	0.000	0.789	0.000
Sand	Pearson						1	−0.633 **	−0.534 **	−0.012	−0.570 **
	Significance							0.000	0.000	0.914	0.000
Silt	Pearson							1	0.205	−0.040	0.264 *
	Significance								0.058	0.713	0.014
SOM	Pearson								1	0.019	0.900 **
	Significance									0.860	0.000
AP	Pearson									1	−0.044
	Significance										0.690
TN	Pearson										1
	Significance										

** Significant correlation at layer 0.01 (double-tailed); * The correlation was significant at 0.05 layers (double-tailed); ‘SOM’ represents soil organic carbon concentration; ‘Clay’ represents the content of soil clay particles; ‘Silt’ represents the content of soil particles; ‘Ele’ represents the altitude; ‘pH’ represents pH of soil.

**Table 4:** Semi-variance parameters of soil element.

Item	Time	Model	Nugget (C_0_)	Partial Sill (C_0_ + C)	Nugget Effect (C_0_/C_0_ + C)	Range (m)	RSS	R^2^
EC	Dry season	gaussian model	16,700	33,410	0.5	92,145	320,000,000	0.448
	Rainy season	spherical model	120	1616	0.926	85,300	2,430,162	0.483
PH	Dry season	exponential model	0.069	0.5	0.862	19,500	0.0689	0.125
	Rainy season	gaussian model	0.379	1.260	0.699	122,802	0.23	0.853
TN	Dry season	gaussian model	0.00042	0.00145	0.710	41,742	0.0000013	0.412
	Rainy season	exponential model	0.00032	0.00166	0.809	22,200	0.0000012	0.092
AP	Dry season	exponential model	2910	7347	0.604	296,700	27,400,000	0.391
	Rainy season	exponential model	95	644.4	0.853	6600	362,579	0.000
SOM	Dry season	exponential model	0.135	0.614	0.780	36,800	0.104	0.484
	Rainy season	exponential model	0.14	0.635	0.780	21,900	0.338	0.051
